# Supplementary material for: A software tool for at-home measurement of sensorimotor adaptation
Source: bioRxiv. 2023 Dec 13:2023.12.12.571359. Preprint. [Version 1] doi: 10.1101/2023.12.12.571359 (PMC10760058; doi:10.1101/2023.12.12.571359)
Supplement: Supplement 2 [file media-2.pdf]

Introduction

This software will allow you to run visuomotor rotation experiments using a laptop or PC. Subjects participate using a touchpad or mouse in order to make “reaching” movements with the mouse cursor. The data is outputted as a text file, and can then be parsed and analyzed using the language of your choice.

In the full version, you can generate full experiment files directly (without the use of a target file), so that subjects only need to download and run a single executable file in order to participate.

What Experiments Can I Run?

This code is able to support the following experiment types/parameters:

- Abrupt perturbations, with or without variance
- Gradual perturbations
- No feedback trials
- Error clamp “perturbation” trials
- Delayed feedback paradigm
- Limit reaction/preparation time paradigm (with automatic reaction time threshold calibration)
- No feedback implicit probe paradigm
- Generalization paradigm
- Savings paradigm
- Interference paradigm
- Spontaneous recovery paradigm
- Multiple targets
- All experiments can have set breaks
- The full code is provided, so that you can add, remove, and edit any features you want so that you can create experiments that are uniquely yours

What Else Is Included?

In addition, you can:

- Include a built-in consent form for biographical data collection
- Include a YouTube video with instructions
    - This video can be embedded within the Java GUI on Windows; otherwise for Mac, a hyperlink is provided instead
- A customizable practice block of trials
- A closing window with links to allow subjects to directly upload their data to a file hosting service (Dropbox, OneDrive, etc) of your choice
- The full code is provided, so that you can add, remove, and edit any features you want so that you can create experiments that are uniquely yours

Requirements

Currently, this code only generates software for Windows and Mac. Each device that runs this software must have Java installed: https://java.com/en/download/

We also recommend using the Eclipse IDE in order to edit and compile this code on your machine: https://www.eclipse.org/downloads/packages/

However, any Java IDE of your choosing should work just as well.

Finally, we also recommend that subjects participate in these experiments using a device that has the capability of playing audio, so that they are able to hear the sounds played during the experiment.

How to Use This Code

Setting up Java + IDE

First, download Java using the following link: https://java.com/en/download/ . Installation for Java should be fairly straightforward - just follow the instructions provided by the installer.

Next, Java code can be edited using any Java Integrated Development Environment (IDE), but we recommend Eclipse. First, download Eclipse and run the installer: https://www.eclipse.org/downloads/packages/ . Choose the first option on the menu that pops up, “Eclipse IDE for Java Developers”:

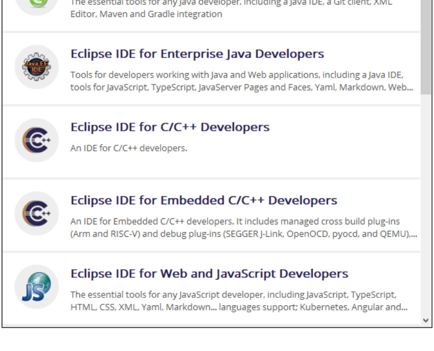

and proceed with the installation.

Once installed, run Eclipse. You will get a message asking to select a directory as your workspace - usually, the default option works fine. Your workspace is the directory in your file system that houses the code, resource files, etc that you will be working with. You can move this folder anywhere within your file system, and browse for it in this window before proceeding.

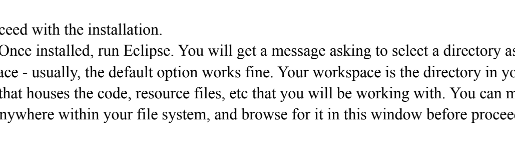

Next, you will see the following Welcome screen. Feel free to “X” out of it:

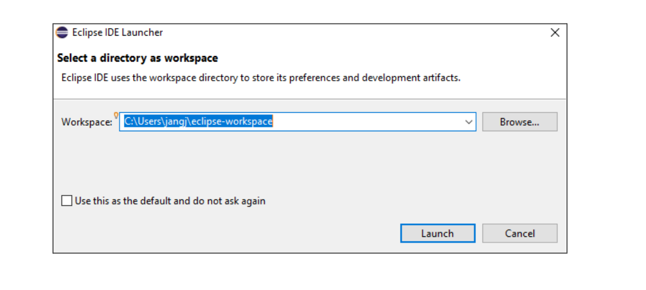

You will then see the main IDE GUI. Before continuing, we recommend adding a “Console” window to the GUI in order to make things easier for yourself in the future. The console will print out errors and print statements, which will help with any debugging that you need to perform. In order to add the “Console,” simply go to Window > Show View > Console. The Console will appear at the bottom of the GUI, and will look like this:

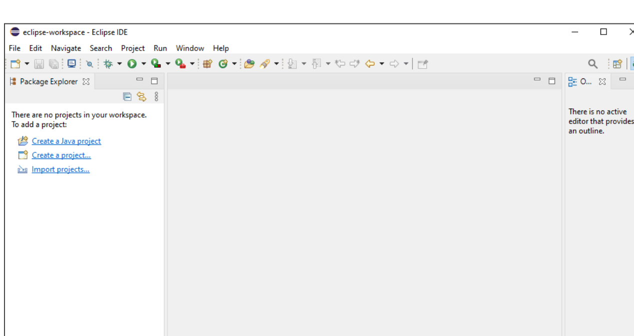

Importing the code

Download the code and its resources (.zip file). Then, in Eclipse, click File > Import > General > Existing Projects into Workspace, and click Next.

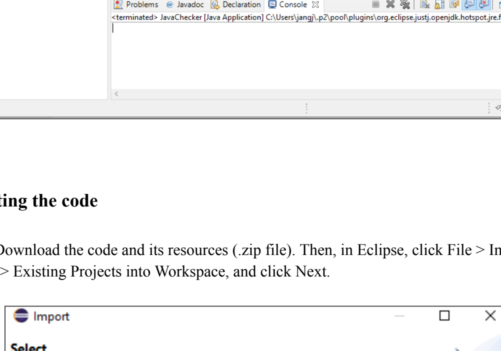

Next, click “Select archive file” and browse within your file system for the .zip file you just downloaded. Click “Finish.”

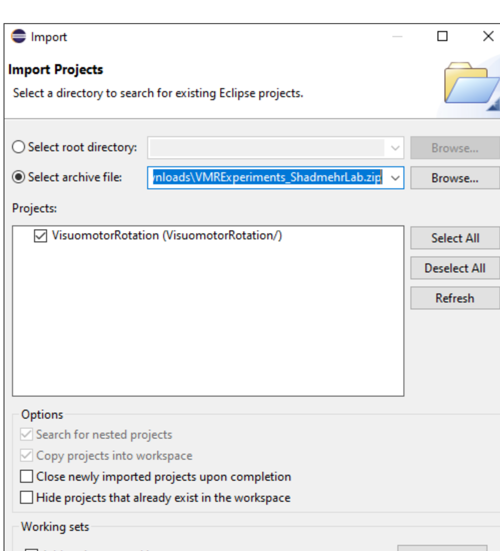

On the left side of the GUI, you should now see the Java project loaded up. This project includes both the code (under “src”), the audio file resources that were used (under “res”), and external .jar files that are needed for the program (under “bin”).

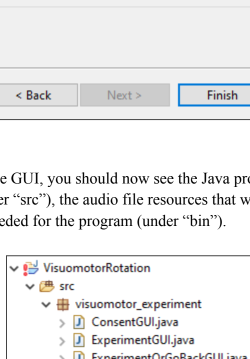

However, we are not done yet. There is a red “!”, indicating an error when we imported the code. This is due to the external .jars failing to migrate properly. To resolve this issue, right click on the parent folder titled “VisuomotorRotation,” and click “Properties.” The following window will open:

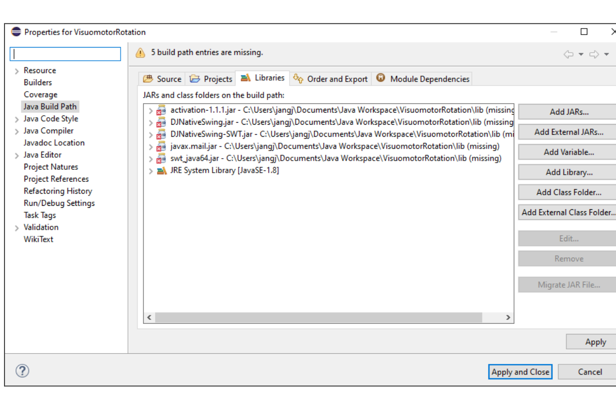

Select all of the .jar with red X’s, and click “Remove” on the right side of the window. Then, click “Add External JARs,” and navigate to the “lib” folder for this project, located within the Eclipse workspace (it is likely that the file explorer will open this folder directly).

Here, select the following files: activation-1.1.1.jar, DJNativeSwing.jar, DJNativeSwing-SWT.jar, and swt_java64.jar. You can ignore the other .jar files for now (by the time you read this manual, it is very likely that I have removed all outdated/deprecated external .jars, so you many only see swt_java32.jar as the .jar that is left over).

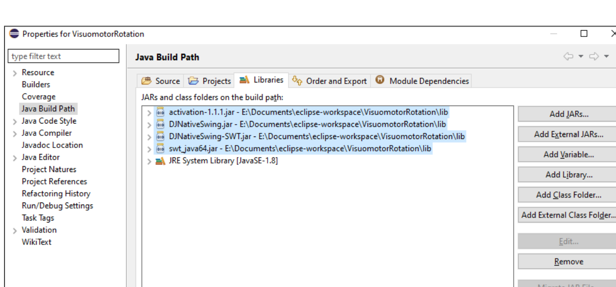

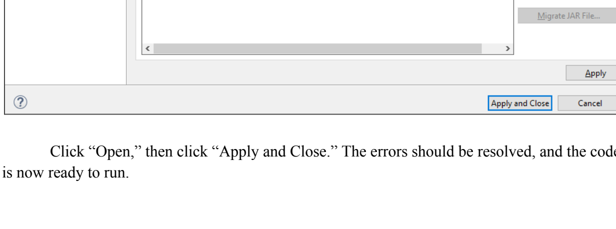

Click “Open,” then click “Apply and Close.” The errors should be resolved, and the code is now ready to run.

Quick detour - how does the code work?

Overview

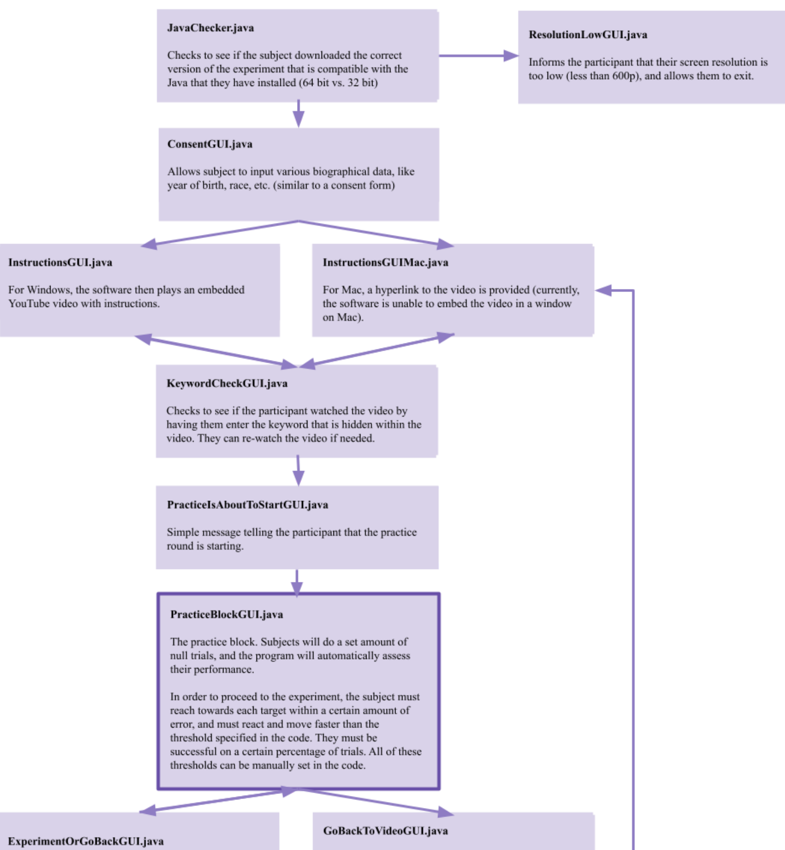

The experiment itself

The code begins by initializing parameters, variables, and GUI components, and designing the GUI window. Arrays containing trial data (target locations, rotation values, etc) are also calculated and generated.

Next are the MouseListener methods, which fire when the mouse enters or exit specific dots in the GUI. These methods allow the code to know when the mouse is currently within either the start or target dot.

Next are the various threads, which act similarly to a finite state machine:

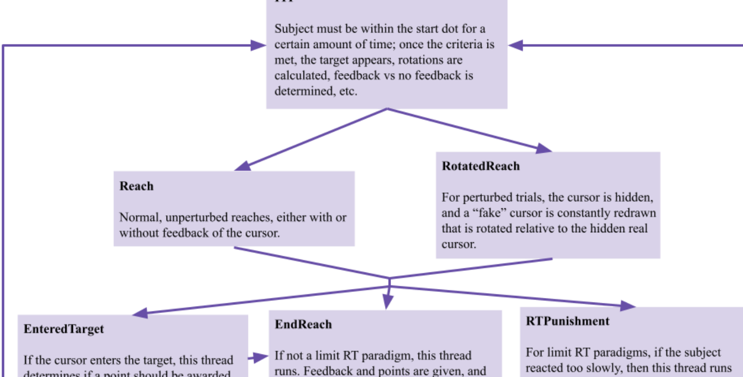

Some key points:

- Rotation perturbations are generated by hiding the real cursor, and constantly redrawing a “fake” cursor on the screen. The coordinates of this fake cursor are constantly calculated by applying a rotation matrix on the coordinates of the hidden real cursor.
- Points are scored when the cursor enters the target at the correct speed. During rotation trials, there is a second invisible and rotated target placed on the GUI with a MouseListener method attached to it. When the invisible, real cursor enters the invisible, rotated target, a point can be scored (and from the subject’s perspective, they see the fake cursor enter a visible target). A point can also be scored if the fake cursor enters the visible target on the screen, ensuring that there is still a way to score a point in case the invisible target doesn’t trigger its MouseListener method when the invisible cursor enters. Flags are added to ensure that only 1 point can be scored on a trial, no matter how many times the cursors enter their targets.

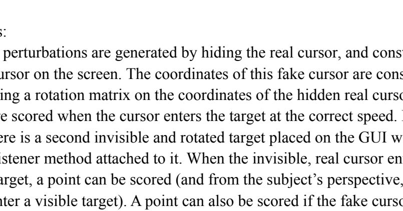

- Once the cursor crosses a specific radius around the start dot, the cursor is frozen in place and the trial ends. To calculate this freeze, the coordinates of the cursor are constantly recorded; once it crosses the radius, the coordinates of the cursor just prior to crossing the radius + the coordinates just after crossing the radius are taken, and the radius crossing point is calculated. The cursor is then displayed at this coordinate.

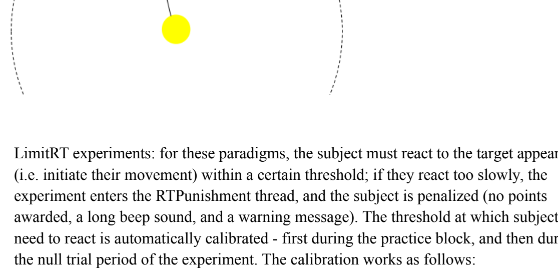

- LimitRT experiments: for these paradigms, the subject must react to the target appearance (i.e. initiate their movement) within a certain threshold; if they react too slowly, the experiment enters the RTPunishment thread, and the subject is penalized (no points awarded, a long beep sound, and a warning message). The threshold at which subjects need to react is automatically calibrated - first during the practice block, and then during the null trial period of the experiment. The calibration works as follows:
    - The first half of the practice block is designated as null trials with no limit RT condition.
    - During the second half, the code looks at a moving window of n trials; if the subject reacts successfully on y% of these n trials, then the reaction time threshold is lowered slightly. Conversely, if they reacted too slowly on many of these trials, then the threshold is increased slightly.
    - There is a lower and upper cap for this threshold, to ensure that the subject doesn’t calibrate to an unreasonably high or low threshold.
    - At the end of the practice block, the threshold value that was calibrated is saved, and then continues to be calibrated during the baseline period of the experiment itself.
    - The size of the moving window (“n”), threshold for success (“y”), threshold increment value, etc. can all be customized in the code.

- An example:

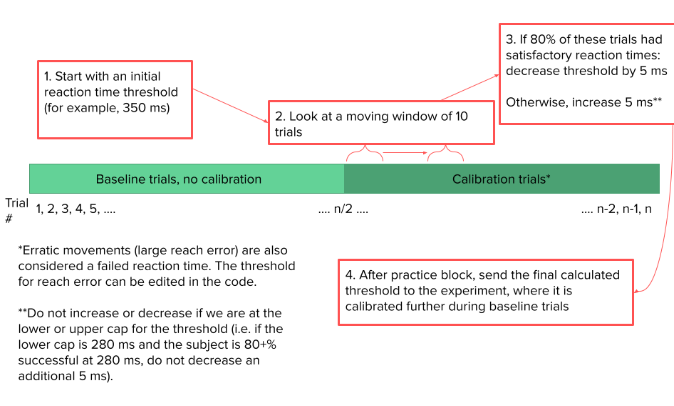

- Note: the code is commented if you are interested in learning more about a feature that is not mentioned in this manual.

Creating an experiment

Before running the code, there are many parameters and variables that you can change to customize the content of your experiment. These are all marked by "TODO" flags so that they are easy to find. They are located in the following .java source files:

1. InstructionsGUI.java and InstructionsGUIMac.java - After creating an instructional video and uploading to YouTube, you can link this video in the Java code so that it is embedded within a GUI window.

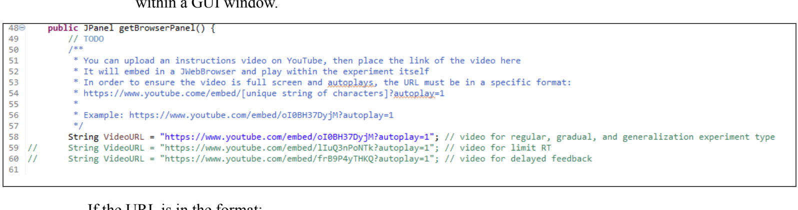

If the URL is in the format:

https://www.youtube.com/embed/[UNIQUE VIDEO CODE]?autoplay=1

then the video will be fullscreen, and start auto playing when the subject reaches this GUI. You can find the unique video code if you watch the video in your browser and look at its URL:

youtube.com/watch?v=adLGHcj_fmA

2. KeywordCheckGUI.java - If you changed the keyword in your video, you can edit it into the code here.
3. PracticeBlockGUI.java and ExperimentGUI.java - Here, you can edit all of the parameters for both the practice block and experiment. A full list of parameters, along with explanations, are provided in the "What Do the Parameters Mean?" section of this manual.
4. ThankYouGUI.java - You can add a link to your file request here. In addition, if there is any paperwork that the subject must fill out for compensation, you can add a link to the .pdf here.

You can now run the code within Eclipse in order to see that it works. In order to run any source file of code, enter that file's code, then press the green play button at the top of the window:

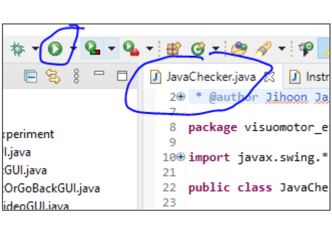

In order to stop the code, you can press the red stop button towards the bottom of the window, above the console:

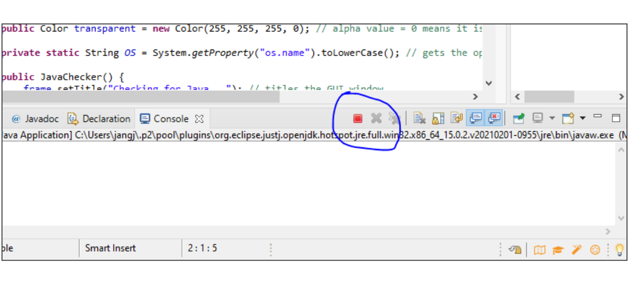

If there are any errors in your code, they will display in the console, which will allow you debug:

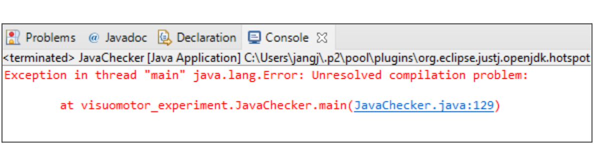

Changing the "bitness" of your experiment

Once you are satisfied with how your experiment runs in the IDE, you can then export it into a runnable executable file. There is no need to make separate versions for Windows or Mac - the program will automatically run different blocks of code depending on the OS of the device.

However, you MAY need to create a 64 bit version (for Mac, and most Windows devices) and a 32 bit version (for a few Windows devices). Most subjects using Windows will install the latest 64 bit version of Java, but subjects using older devices or Java versions may have 32-bit and will thus require a 32 bit experiment. In order to change the bitness of the experiment, do the following:

1. In JavaChecker.java, change the String variable "bit" to either "64" or "32" (the version you are currently in the process of creating).

```
public static String bit = "64";
```

2. Then, right click on the experiment project in the menu on the left side:

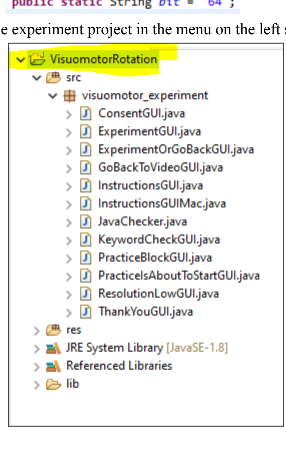

and click "Properties."

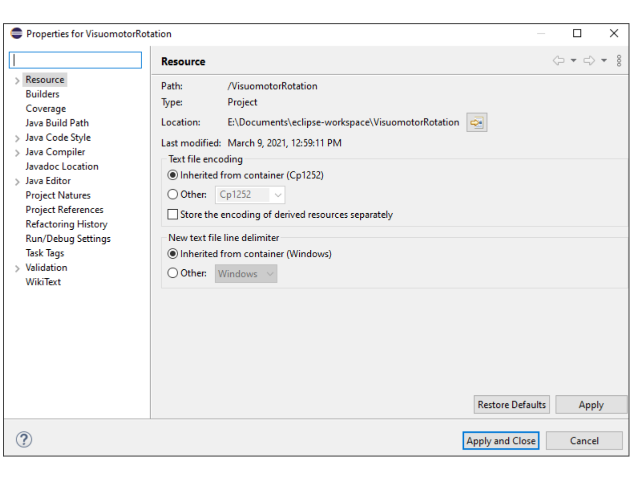

3. Navigate to "Java Build Path," and then to the "Libraries" tab.

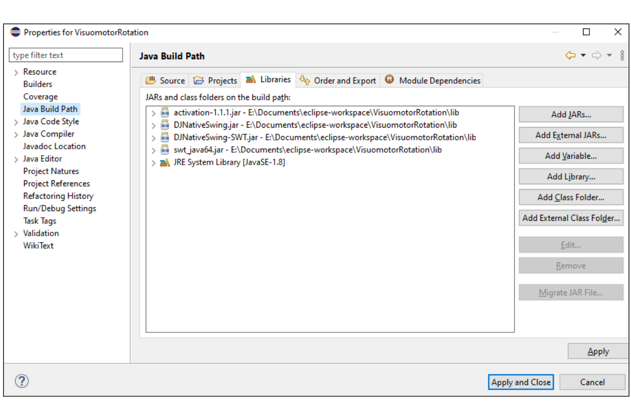

4. If you are making a 32 bit version, click "swt_java64.jar" and click "Remove" on the right side of the screen. If making a 64 bit version, do the opposite.

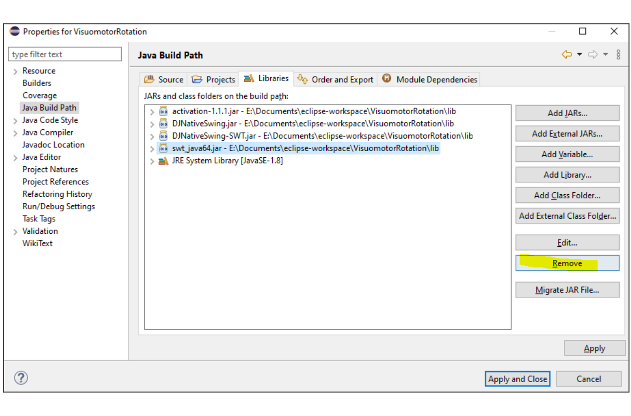

5. Then, click "Add External JARs," navigate to the "lib" folder, and select the correct swt_java file (if making a 32 bit program, choose swt_java32.jar, and vice versa).

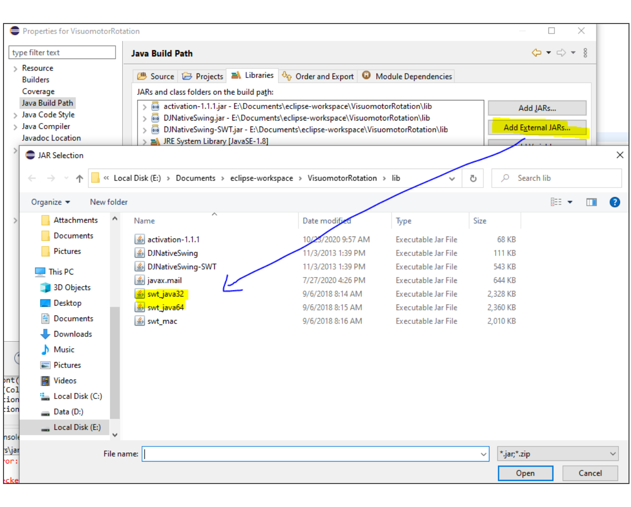

6. You can then press "Apply and Close," and make sure one last time that the bitness of the swt_java file AND the String variable from step 1 both match.

Exporting the experiment

In order to export the program as an executable file, right click on the project in the menu on the left side, and click "Export."

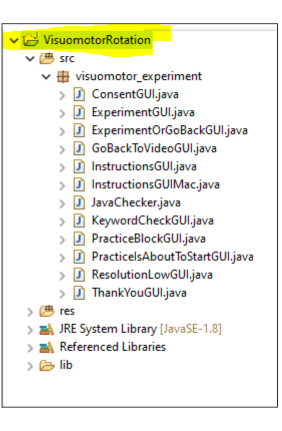

Select the "Java" folder, choose "Runnable JAR file," and click "Next."

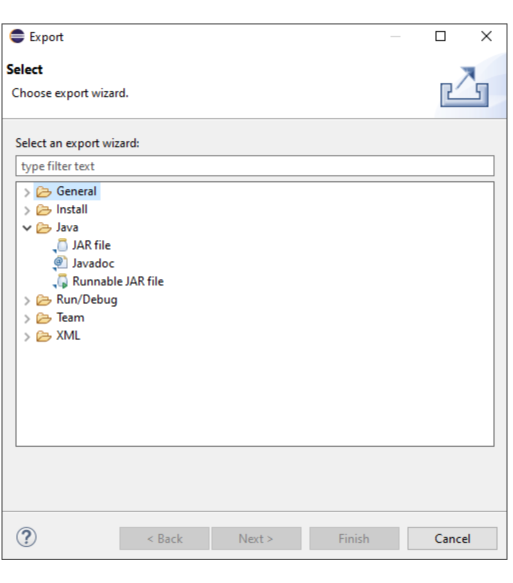

Under "Launch configuration," choose "JavaChecker." This will ensure that the entry point of the executable (the first block of code that is run) when the subject double clicks on the file is the JavaChecker code.

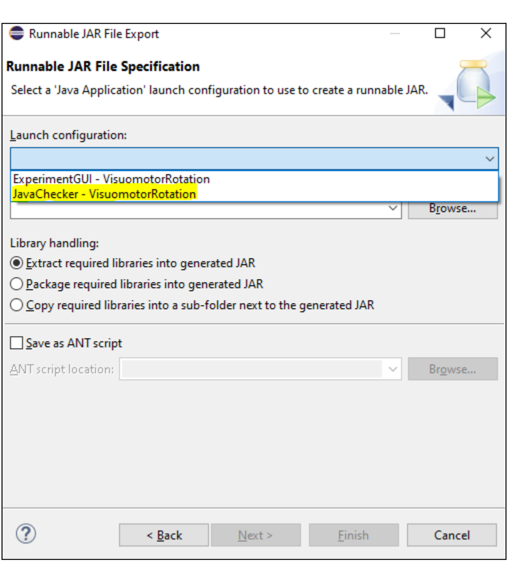

You can then click "Browse" to choose an Export destination in your file system, as well as name your executable .jar file.

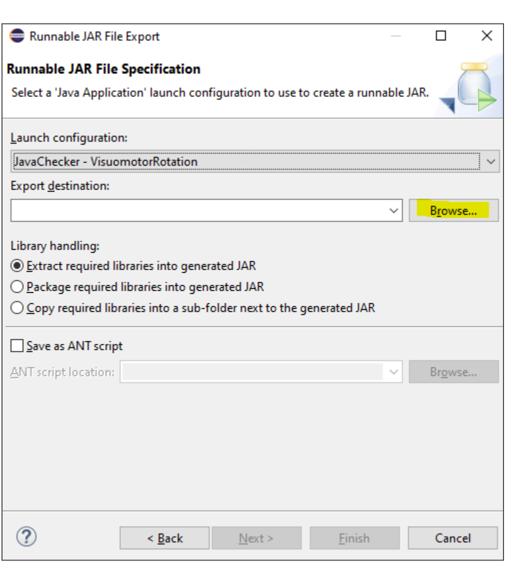

Press "Finish," and the executable .jar file will generate.

If you are testing something out and want to view error messages while running the executable file, you can open it via the command line (not double clicking). Error messages will print in the command line. Navigate to the folder with the executable file and enter the following command:

```
java -jar [NAME OF EXECUTABLE].jar
```

Analyzing data

Every time the experiment runs, it will output a unique text file containing the data. Using the programming language of your choice, you can analyze this data by loading in the text file and parsing it. For example, in Matlab this can be achieved using the "regexp" function to look for specific regular expressions.

What Do the Parameters Mean?

All values should be integers unless otherwise stated.

- **num_baseline** - Specifies the number of baseline (null) trials in the experiment.
- **num_rotation** - Specifies the number of perturbation trials in the first half of the experiment. If not running savings, interference, or spontaneous recovery, then the number of trials indicated here is the total number of perturbation trials in the experiment.
- **num_washout** - Specifies the number of washout trials in the experiment. Set this value to 0 if not running savings, interference, or spontaneous recovery.
- **num_rotation_2** - Specifies the number of perturbation trials in the second half of the experiment, if running saving/interference/spontaneous recovery.
- **num_no_feedback** - Specifies the number of no feedback trials at the end of the task.

Note: the experiment assumes that the order of trials for non-generalization experiments is baseline -> rotation -> washout -> rotation_2 -> no_feedback. If you wanted to, for example, run a simple abrupt perturbation experiment with no feedback trials at the end, then just set num_washout and num_rotation_2 equal to 0. You can't, however, have washout occur after rotation_2.

- **num_generalization_rotation** - Specifies the number of perturbation trials that are present in each cycle of generalization.
- **num_generalization_NF** - Specifies the number of no feedback probes during each cycle of generalization.
- **num_generalization_cycles** - Specifies the number of generalization cycles.
- **angle_range_1** - Specifies the lower bound angle for generalization no feedback probes. This value should be a double (i.e. has a decimal point), and is in degrees.
- **angle_range_2** - Specifies the upper bound angle for generalization no feedback probes. This value should be a double (i.e. has a decimal point), and is in degrees.

Note: for generalization tasks, the structure of trials is num_baseline -> num_rotation -> (num_generalization_rotation + num_generalization_NF)*num_generalization_cycles. During the rotation portion of the cycle, the task is identical to the perturbation block - subjects will reach towards targets with a standard VMR perturbation. During the no feedback portion of the cycle - for every no feedback probe trial, a target will randomly appear at evenly spaced locations between angle_range_1 and angle_range_2. (For example, if we set num_generalization_NF = 3, angle_range_1 = 70, and angle_range_2 = 90, then during this portion of the cycle, we would have three reaches to three targets: one appearing at 70 degrees, one at 80, and one at 90.)

- **num_targets** - Number of targets in the experiment, evenly spaced. The order of the targets are randomized during the experiment.
- **ITI** - The amount of time that the subject must spend on the start dot before the trial starts. This value should be a double (i.e. has a decimal point), and is in ms.
- **minimum_reach_time** - The minimum amount of time that the subject must spend during their reach. If they reach faster than this, then they are penalized for moving too fast. Measured in ms.
- **maximum_reach_time** - The maximum amount of time that the subject must spend during their reach. If they reach slower than this, then they are penalized for moving too slow. Measured in ms.
- **warning_reach_time** - If they reach slower than even this, then they are penalized for moving too slow, and also receive a warning on the screen. Measured in ms.
- **non_limit_rt_threshold** - During a non limit reaction/preparation time trial, if a subject reacts to the trial slower than this threshold, then they are penalized for moving too slow, and also receive a warning on the screen. Measured in ms.
- **angle_mean -** Specifies the angle of perturbation during the num_rotation block. If running a gradual paradigm, this is the angle that the experiment plateaus at. A negative angle means the cursor is rotated counterclockwise. This value should be a double (i.e. has a decimal point), and is in degrees.
- **angle_std** - Specifies the standard deviation of the perturbation. If this value is non zero, the experiment will randomly generate perturbations from a normal distribution using the programmed value for angle_mean as the mean, this value as the standard deviation. This value should be a double (i.e. has a decimal point), and is in degrees.
- **angle_mean_2 -** Specifies the angle of perturbation during the num_rotation_2 block. If running savings, make sure that angle_mean = angle_mean_2. This value should be a double (i.e. has a decimal point), and is in degrees.
- **angle_std_2** - See entry for "angle_std"
- **is_delayed_feedback -** Integer flag to indicate if this experiment should run a delayed feedback paradigm. Setting it to "1" means it is delayed feedback; otherwise, set it to 0.
- **delayed_feedback_delay** - The duration of the delay before feedback is shown during delayed feedback trials. Measured in ms.
- **is_limit_rt -** Integer flag to indicate if this experiment should run a limit reaction/preparation time paradigm. Setting it to "1" means it is limit RT; otherwise, set it to 0.
- **rt_threshold -** During a limit reaction time paradigm, if the subject reacts slower than this threshold, then they fail the trial and are "punished" with a warning message and long tone. This value is automatically calibrated during the baseline period of the experiment in order to ensure that subjects are able to perform the task with some success. The value you place here in the target is the initial starting point of calibration. Measured in ms.
- **angle_threshold -** During calibration, this threshold determines the angle at which the subject must move within in order for that calibration trial to be successful. For example, if this threshold is set at 30 degrees, but the subject reaches towards the target at 40 degrees (either clockwise or counterclockwise to the target), then the trial is counted a failure, even if the subject reacted quickly enough. This field should be a positive double, measured in degrees.
- **lower_rt_cap** - The lower bound for RT threshold calibration; rt_threshold can not go lower than this value. Measured in ms.
- **upper_rt_cap** - The upper bound for RT threshold calibration; rt_threshold can not go higher than this value. Measured in ms.
- **RT_calibration_window -** The length of the moving window that the calibration algorithm uses. Measured in number of trials (i.e. a value of 10 means that the algorithm looks at a moving window of 10 trials during baseline).
- **RT_calibration_threshold -** The threshold of success that must be attained within a moving window in order to decrease the rt_threshold value. Otherwise, this value will increase. Must be a double, and a value between 0 and 1.
- **RT_calibration_increment** - The amount in which the rt_threshold value is changed depending on the success or failure of the moving window. Measured in ms.

Note: The calibration algorithm works as follows:
During baseline, subjects will reach towards targets in a null condition. Within a moving window of trials defined by RT_calibration_window, each individual trial will be classified as either pass ("1") or fail ("0"). In order to pass, the reach must be within the threshold defined by angle_threshold, and the subject must react faster than the value currently assigned to rt_threshold. Then, the trials in the moving window are assessed to see if the percentage of successes is greater than or lower than the threshold defined by RT_calibration_threshold. If success is greater, then rt_threshold is lowered by a value equal to RT_calibration_increment, and vice versa. If a subject reaches either lower_rt_cap or upper_rt_cap, then rt_threshold cannot be incremented further above or below these caps, even if the moving window is successful or not, respectively.

- **is_no_feedback_implicit_probe -** Integer flag to indicate if this experiment should run a no feedback implicit probe paradigm. Turning this flag on means that before the no feedback block of trials, the experiment pauses and displays a message (with a voiceover) that instructs subjects to reach straight to the target. Setting it to "1" means it is an implicit probe paradigm; otherwise, set it to 0.
- **is_EC -** Integer flag to indicate if this experiment should run an error clamp paradigm, where reach movements during the rotation period are clamped at the angles specified by angle_mean and angle_mean_2. Setting it to "1" means it is an error clamp paradigm; otherwise, set it to 0.
- **is_gradual -** Integer flag to indicate if the perturbation is gradual, rather than abrupt. Setting it to "1" means it is a gradual paradigm; otherwise, set it to 0.
- **num_plateau -** Specifies the number of plateau trials that will be present after the perturbation is gradually ramped up to the maximum value specified by angle_mean.

Note: If running a gradual paradigm, the rotation block is constructed as follows: the total number of perturbation trials is encoded by num_rotation. Then, this block is divided into two parts: the gradual ramping up, and the plateau period. The number of plateau trials is specified by num_plateau; from there, num_gradual is simply num_rotation - num_plateau.

- **is_generalization -** Integer flag to indicate if this is a generalization paradigm. Setting it to "1" means it is a generalization paradigm; otherwise, set it to 0.
- **post_reach_delay -** Determines the amount of delay at the end of each trial. After feedback is given, the experiment momentarily freezes in order to give the subject time to process their performance. Measured in ms.
- **is_savings -** Integer flag to indicate if this is a savings paradigm. Setting it to "1" means it is a savings paradigm; otherwise, set it to 0.
- **is_interference -** Integer flag to indicate if this is an interference paradigm. Setting it to "1" means it is an interference paradigm; otherwise, set it to 0.
- **is_spontaneous_recovery -** Integer flag to indicate if this is a spontaneous recovery paradigm. Setting it to "1" means it is a spontaneous recovery paradigm; otherwise, set it to 0.

Note: In order to generate a spontaneous recovery paradigm, you can utilize the following structure: baseline -> rotation -> rotation_2 -> no feedback. Set num_washout = 0.

- **set_break_length -** The length of set breaks during the experiment. Measured in ms.
- **set_break_trial_nums** - Specifies the trials at which set breaks occur. For example, if trial number 5 is indicated here, then the set break happens between trial 4 and trial 5. For multiple set breaks, separate trial numbers using commas (e.g. 40, 120, 260)

These last two parameters are only found in the practice block:

- **angle_threshold** - Defines the threshold of reach error for a movement to determine if it is erratic or not. This field should be a double.
- **practice_percent_correct_threshold** - Defines the percent of trials during practice that must be acceptable (reacted quickly, reached quickly, not erratic) to proceed. This field should be a double.
