# Supplementary material for: A software tool for at-home measurement of sensorimotor adaptation
Source: bioRxiv. 2023 Dec 13:2023.12.12.571359. Preprint. [Version 1] doi: 10.1101/2023.12.12.571359 (PMC10760058; doi:10.1101/2023.12.12.571359)
Supplement: Supplement 3 [file NIHPP2023.12.12.571359V1-supplement-3.pdf]

bioRxiv preprint doi: https://doi.org/10.1101/2023.12.12.571359; this version posted December 13, 2023. The copyright holder for this preprint (which was not certified by peer review) is the author/funder, who has granted bioRxiv a license to display the preprint in perpetuity. It is made available under aCC-BY 4.0 International license.

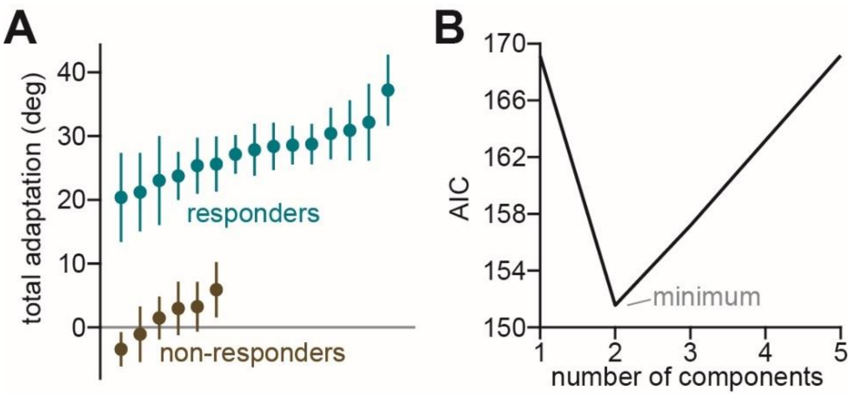

Figure 3-Supplement 1. Two subgroups in the response to delayed error feedback. **A.** We calculated the movement angle over the last 10 rotation epochs in Experiment 5 (n=21). Visually, two subgroups were apparent in the data. **B.** To rigorously test whether participants fell into two groups, we fit a Gaussian mixture model, and varied the number of components between 1 and 5. We selected the model with the smallest AIC value (2 components).
